# How neurons migrate: a dynamic in-silico model of neuronal migration in the developing cortex

**DOI:** 10.1186/1752-0509-5-154

**Published:** 2011-09-30

**Authors:** Yaki Setty, Chih-Chun Chen, Maria Secrier, Nikita Skoblov, Dimitrios Kalamatianos, Stephen Emmott

**Affiliations:** 1 Computational Science Laboratory, Microsoft Research, Cambridge, CB3 0FB, UK; 2 Department of Computer Science and Applied Mathematics, Weizmann Institute of Science, Rehovot, Israel; 3 Centre National de la Recherche Scientifique, Paris, France; 4 European Molecular Biology Laboratory, Heidelberg, Germany; 5 The Faculty of Computational Mathematics and Cybernetics, Moscow State University, Moscow, Russia; 6 The Hamilton Institute, National University of Ireland, Maynooth, Ireland

## Abstract

**Background:**

Neuronal migration, the process by which neurons migrate from their place of origin to their final position in the brain, is a central process for normal brain development and function. Advances in experimental techniques have revealed much about many of the molecular components involved in this process. Notwithstanding these advances, how the molecular machinery works together to govern the migration process has yet to be fully understood. Here we present a computational model of neuronal migration, in which four key molecular entities, Lis1, DCX, Reelin and GABA, form a molecular program that mediates the migration process.

**Results:**

The model simulated the dynamic migration process, consistent with in-vivo observations of morphological, cellular and population-level phenomena. Specifically, the model reproduced migration phases, cellular dynamics and population distributions that concur with experimental observations in normal neuronal development. We tested the model under reduced activity of Lis1 and DCX and found an aberrant development similar to observations in Lis1 and DCX silencing expression experiments. Analysis of the model gave rise to unforeseen insights that could guide future experimental study. Specifically: (1) the model revealed the possibility that under conditions of Lis1 reduced expression, neurons experience an oscillatory neuron-glial association prior to the multipolar stage; and (2) we hypothesized that observed morphology variations in rats and mice may be explained by a single difference in the way that Lis1 and DCX stimulate bipolar motility. From this we make the following predictions: (1) under reduced Lis1 and enhanced DCX expression, we predict a reduced bipolar migration in rats, and (2) under enhanced DCX expression in mice we predict a normal or a higher bipolar migration.

**Conclusions:**

We present here a system-wide computational model of neuronal migration that integrates theory and data within a precise, testable framework. Our model accounts for a range of observable behaviors and affords a computational framework to study aspects of neuronal migration as a complex process that is driven by a relatively simple molecular program. Analysis of the model generated new hypotheses and yet unobserved phenomena that may guide future experimental studies. This paper thus reports a first step toward a comprehensive in-silico model of neuronal migration.

## Background

Neuronal migration is a highly dynamic process that is essential for the normal development and function of the mammalian brain. The migration process is regulated by cell-extrinsic signaling pathways and cell-intrinsic regulation and implicates numerous molecules that synergistically guide neuron motility. The experimental data have revealed that the process initiates with proliferation of neuroblasts from the progenitor glial cells at the ventricular zone (VZ). The neuroblasts migrate radially toward the subventricular zone (SVZ) along the glial fiber, which serves as a their scaffold [1]. On exit from the VZ, neuroblasts adopt a multipolar migration stage in which they disassociate from the glial fiber and migrate independently through the intermediate zone (IZ) [1,2]. As the multipolar neurons approach the cortical plate (CP), they enter a bipolar migration stage and re-associate with the glial fibers. On entry into the CP zone, the migrating neurons dissociate from the fiber and accumulate in a layer above that formed by their predecessors, such that layers of progressively younger neurons are found from interior to surface [3,4].

Advances in experimental techniques and genetic studies have identified key molecular components that are implicated in the migration process. Yet, how these components act in concert to mediate neuronal migration at the system-level is not fully understood. Key outstanding questions that remain unsolved include how neurons interpret guidance cues, what are the specific molecular mechanisms that guide migration, and how neurons coordinate interactions between different signaling pathways. Theoretical study may afford a platform to explore how the different components orchestrate the migration process as a whole. Because of the high degree of molecular complexity governing neuronal migration simplification is required. This has been constructively demonstrated in previous studies that have categorized the molecular entities into functional classes [5] or mechanical instructions [6]. We have adopted similar simplifications in our modeling study.

The computational model we present here integrates isolated subcomponents of the migration process into a dynamic system-level simulation of the process. We focused on the interplay between four molecular entities that have been found to be crucial for normal migration: two extracellular signaling cues, GABA neurotransmitter and Reelin protein, and two intracellular regulators, the Lissencephaly1 (LIS1) and Doublecortin (DCX) proteins. We defined the following functionalities for GABA, Reelin, Lis1 and DCX: (1) GABA neurotransmitter has a chemotactic role and a chemokinetic effect on migrating neurons. Experimental observations have found that GABA influences migrating cortical neurons [1,7]. We, therefore, defined GABA to act as a chemoattractant that provides, together with the glial fiber, guidance cues for migrating neurons. (2) Reelin is implicated in the maintenance of the radial scaffold [8] and neuron polarization as well as in stabilizing the leading process and promoting the final soma translocation [9]. Reelin is secreted by Cajal-Retzius cells located at the pial surface and is predominantly expressed in the CP [10]. In addition, Reelin was found to induce dissociation of neurons from the glial fiber [11]. Therefore, in our model Reelin serves as a local stop signal for migrating neurons that enter the CP [3,5]. (3) The intracellular regulators, Lis1 and DCX, mediate neuronal polarization by stabilizing microtubules [12] and are implicated in the regulation of leading process branching and dynamics [13]. In addition, these regulators play a role in the proliferation of neural progenitors [14] and have also been attributed with unknown cell-nonautonomous functions [15]. Silencing expression of Lis1 and DCX by RNA inhibition has been shown to arrest normal migration and to result in aberrant migration with a distinct pattern [1,16-18]. Thus, in the model, we specified Lis1 and DCX as regulators that stimulate and guide neuron motility and timing. (4) For modeling purposes we assumed that the association with the glial fiber is controlled via the (direct or indirect) interaction of the motility regulators, Lis1 and DCX, with Astrotactin, which was found to mediate neuron movement along the glial fiber [19]. We are not, however, aware of any experimental findings that support or refute this assumption.

Our model incorporates only a subset of the rich genetic knowledge. The molecular entities, even in this compact subset, have a variety of complex functions and are implicated in various signaling pathways and mechanisms. Nevertheless, we found that the model simulated aspects of morphology, distribution and cellular dynamics, consistent with experimental observations. Furthermore, the simulations under reduced Lis1 and DCX activity concurred with observations from Lis1 and DCX silencing experiments using RNA interference.

The modeling process and the model analysis were informative and served as a platform to study the neuronal migration at the system-level rather than isolated components. The dynamic nature of the simulation generated behaviors that were not explicitly programmed into the model. These emergent behaviors are properties of the system as a whole and can only be explored as such. The simulations give rise to insights that may guide future experimental trial. Specifically, the model pointed to the possibility that under Lis1 reduced expression conditions, neurons experience an oscillatory neuron-glial association prior to the multipolar stage. Additionally, using the model we studied bipolar migration under altered expression levels of the key regulators Lis1 and DCX in rats and mice. The analysis led us to hypothesize that the observed morphology variations in these organisms may be explained by a single difference in the way the Lis1 and DCX stimulate bipolar motility. This hypothesis helped us to predict the bipolar morphology under yet untested expression levels of these regulators. We predict that cortical migration in rats under reduced expression of Lis1 and overexpression of DCX will reduce the bipolar migration, in contrast with the normal morphology observed in mice. Additionally, we suggest that bipolar morphology in mice under DCX overexpression will give rise to normal or higher bipolar morphology similar to the bipolar morphology observed in rats under similar conditions.

## Results and Discussion

### Model construction and execution

**Figure 1:**
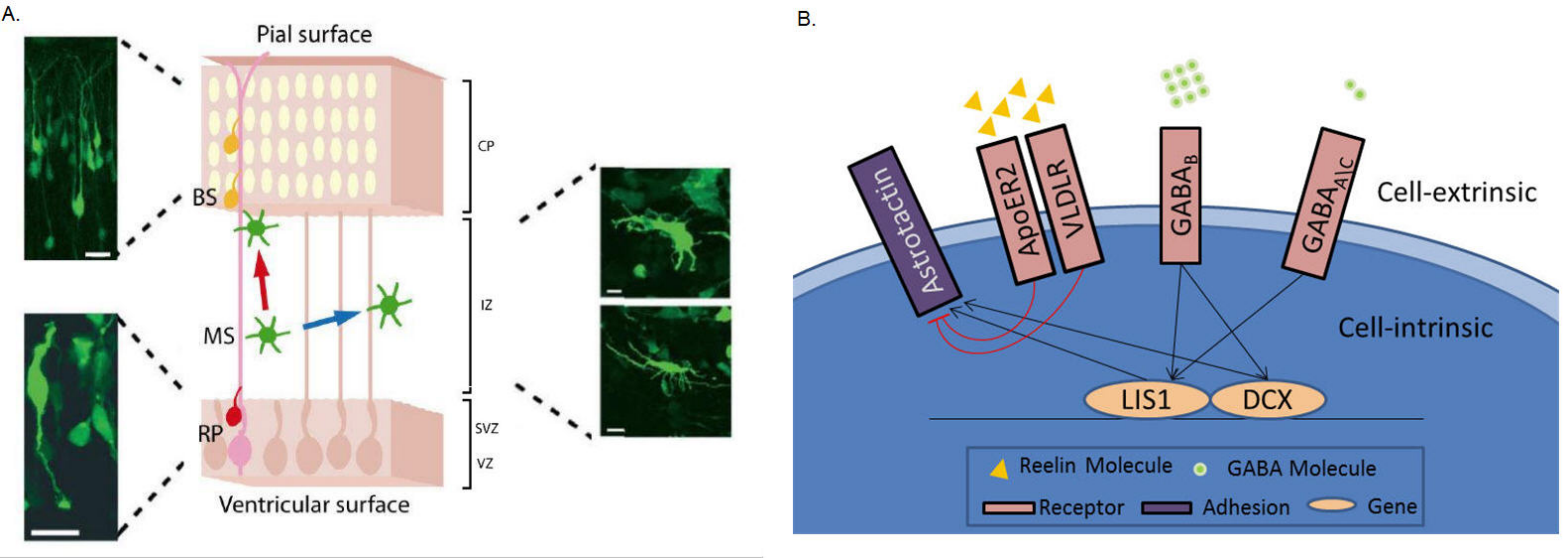
**Illustration of the neuronal migration process and the simplified set of rules in the in-silico model**. A: An illustration of neuronal migration (reproduced with permission from [1]). Radial progenitor glial cells (RP; designated by a pink cell with a fiber) proliferate to give rise to neuroblasts (PN; designated by a red cell). Neuroblasts migrate guided by the glial fiber at the ventricular zone and subventricular zone (VZ/SVZ). Neuroblasts adopt the multipolar stage (MS; designated by a green star-like cell) when entering the intermediate zone (IZ) and migrate independently from the glial fiber (on both X and Y directions). In the cortical plate (CP) neurons adopt bipolar state (designated in yellow), associate with the glial fiber and accumulate on top of the pile. The histology images correspond to the various migration stages of the migrating neurons: neuroblasts (bottom-left), multipolar stage neurons (right) and bipolar neurons (top-left). B: Description of the interaction scheme in the model: GABA_A _detects low concentrations of GABA neurotransmitter in the cortex, but maintains its activity for short periods. GABA_A _in its active form promotes the activity of Lis1. GABA_B _requires higher concentrations of the neurotransmitter. Active GABA_B _promotes both Lis1 and DCX independently. Active Lis1 and active DCX promote Astrotactin adhesion with the glial fiber. The two Reelin Receptors (VLDLR and ApoER2) act as one unit that detects Reelin to reduce the ability of Astrotactin to adhere with the glial fiber one activated.

To construct a model that reproduces the distinct stages of the migration process (as illustrated in Figure 1A), we specified the interaction scheme shown in Figure 1B (see Methods for details of model formulation, construction and simulation). The model makes a number of simplifying assumptions: First, interactions occur between five extracellular mediators (GABA_A_, GABA_B_, ApoER2, VLDLR and Astrotactin) that regulate two intracellular regulators (Lis1, DCX). A decreasing GABA concentration gradient exists from the CP toward the VZ [1,7] reflected by a trend in Reelin activity over the CP [3,20]. Our second assumption is that once the GABA_A _receptor of a neuroblast detects GABA, it promotes Lis1 activity to maintain the glial fiber association [21]. This assumption is based on previously published possible relationships between GABA and Lis1 in *C. elegans *[22], and in-vitro experiments that suggested that GABA induces dissociated neurons to migrate from the VZ to the IZ [2] via GABA_A _receptor, which possibly plays role in neuron movement [7]. Third, we specify that GABA_A _activity is reduced to a level below that required to promote Lis1. Consequently, neurons enter the multipolar stage and disassociate from the glial fiber to migrate randomly in both X and Y directions [1,23]. Fourth, when GABA_B _detects higher concentrations of GABA, it activates Lis1 and DCX to re-associate with the fiber and to enter the bipolar stage. This is based on observations that GABA_B _receptors promote migration from the IZ to the CP [1,7], and on a suggested role for DCX in stabilizing neurons association with the glial fiber [2]. Finally, bipolar neurons migrate radially until the Reelin receptors (i.e., ApoER2 and VLDLR) detect and bind to Reelin (i.e., the stop signal) and inhibit neuron's adherence ability to the glial fiber. In turn, neurons dissociate from the glial fiber and accumulate on the next layer in the CP [4,20]. To simplify the complex mechanism by which Lis1, DCX and Reelin regulate the association with the fiber, we defined for modeling purposes that these molecular components mediate the adherence ability of neurons to the fiber through interaction with the Astrotactin protein that mediates the neuron-glial adhesion ligand (although no experimental evidence supports this assumption) [19].

The model is seeded by 23 radial progenitor glial cells in the simulated cortical cross-section. The progenitor glial cells proliferate and give rise to neuroblasts. Each of the neurons executes the same molecular program to determine its migration behavior over time. For example, a neuroblast at the SVZ activates GABA_A _receptors in response to GABA concentrations in its vicinity (see detailed description in Methods).

### The model recapitulates complex system-level neuronal migration

**Figure 2:**
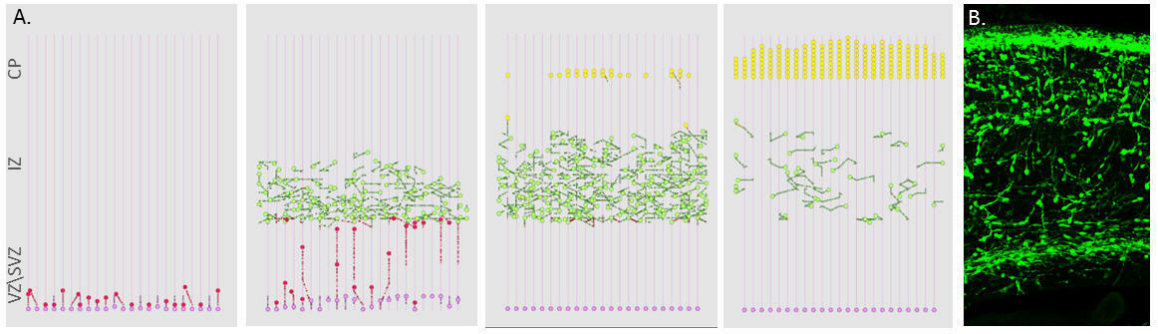
**Normal neuronal migration in the mammalian cortex**. A. Snapshots of the neuronal migration simulation. Radial progenitor glial cells (pink) proliferate asymmetrically giving rise to neuroblasts (red). Neuroblasts migrate radially on the glial fiber. Neuroblasts adopt a multipolar stage (green) and migrate independently from the glial fiber. Multipolar neurons re-associate with the glial fiber, enter the bipolar migration (yellow) and accumulate on the pile surface. B. Histology of the mouse cortex at embryonic day 16 (reproduced with permission from [53]).

Model executions provided a dynamic representation of neuronal migration in the cortex (Figure 2A and the movie in Additional File 1). Neuroblasts (red) proliferated from the glial mother cells (pink) and migrated along the glial fiber in the VZ/SVZ radially toward the IZ. On entry to the IZ, neurons adopt the multipolar stage and migrate randomly both horizontally and vertically independent from the glial fiber (green). Finally, neurons adopted a bipolar state (yellow) and re-associated to a glial fiber. The neurons maintained glial-guided radial migration when locating from the IZ to the CP. Having entered the CP, they continued vertical bipolar migration before dissociating from the fiber in response to Reelin and subsequently accumulating in layers at the CP.

**Figure 3:**
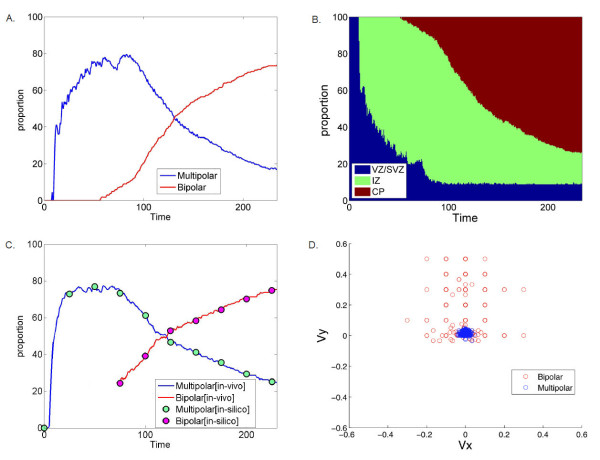
**Cellular and population dynamics in neural migration simulations**. A. Distribution of multipolar (blue) and bipolar (red) neurons over time (averaged over 10 different simulations). B. Zone occupancy as function of time (VZ/SVZ blue), IZ (green) and CP (red) (averaged over 10 different simulations). C: Migration stage over time as determined by the path formed by neuronal movement (Solid line: mathematical analysis of the migration data using in-vivo parameters (from [24]) multipolar population (blue) bipolar population (red). Circles: mathematical analysis of the migration data using in-silico parameters: multipolar population (green) and bipolar population (purple). D: Migration stage as a function of velocity in X and Y directions, multipolar (blue) and bipolar (red).

Simulation results and experimental observations of neuronal population distribution were in general agreement. The model, consistent with histology (Figure 2B), generated a narrow band of progenitor glial cells at the VZ/SVZ boundary, a sparse occupancy of neurons in the IZ and neuron accumulation at the cortical surface (CP). The multipolar population dominated the earlier stages of migration (blue) and gradually adopted a bipolar fate (red) (Figure 3A). Similarly, the occupancy of neurons in the different zones of the simulated and experimental cortex cross-section showed a gradual migration over time. At the early developmental stages the majority of neurons positioned at the VZ/SVZ (blue). As the simulation advances the neurons rapidly move to the IZ (green) and then accumulate at the CP (red) (Figure 3B). At the end of the simulation 77% of the neuronal population was bipolar, 14% multipolar and 9% radial glial cells. The cortex occupancy in the simulated cross-section showed 9% of cells occupying the VZ/SVZ zones, 11% the IZ and 80% the CP. Experimental observations report a similar population distribution in the mouse cortex [24]: 75% bipolar neurons, 10% multipolar and 10% glial cell (5% unclassified), with zone occupancies of 7%, 15% and 78% at the ZV/SVZ, IZ and CP zones respectively.

Neuron behavior differs between different migration stages. Multipolar neurons actively explore their environment for directional cues and migrate independently from the glial fiber, moving randomly in the X and Y directions [2,25]. In contrast, bipolar migration is glial-guided and is characterized by a radial movement biased in the Y direction. Previous studies [17,25] suggested that it is possible to distinguish between multipolar neurons and bipolar neurons based on the path they follow and their respective velocities. To test the relationship between migration stage and path formation, we applied a previously employed mathematical technique that distinguishes between multipolar and bipolar neurons by path formation in-vitro [25]. The analysis revealed a mean square error of less than 10% between the classifications of neurons in the model using in-vivo and in-silico parameters (Figure 3C). Additionally, we analyzed relationships between the velocity of the neurons and their migration stage by plotting migration stage as a function of the horizontal and vertical components of the migration velocity (Figure 3D). Neurons in the multipolar stage were found to have a low velocity in both directions (blue), while neurons in the bipolar stage had typically a higher, predominantly Y-directional, velocity (red). This analysis concurs with experimental findings that multipolar neurons remain closer to their origin, whereas bipolar neurons are found at a significant distance from their origin [17,25].

### Abnormal GABA-Lis1 interaction anticipates aberrant migration as observed in Lis1 RNAi studies

Lis1 regulator has a complex function in the migration and plays a role in various signaling pathways and mechanisms. One function that is attributed to Lis1 is stabilizing neuron association with the glial fiber [2]. Normal migration in Lis1 RNAi mice is arrested in its early stages [1,2,21] and displays accumulation of multipolar neurons at the SVZ and lower IZ [21]. In our model, Lis1 acts as a motogen that stimulates and guides the motility of neurons along the glial fiber in response to GABA concentrations. Thus, in Lis1 reduced activity simulations, neuroblasts that detect GABA concentrations did not express Lis1 normally. Under these conditions, neuroblasts expressed Lis1 only at the random basal level embedded in the molecular program.

**Figure 4:**
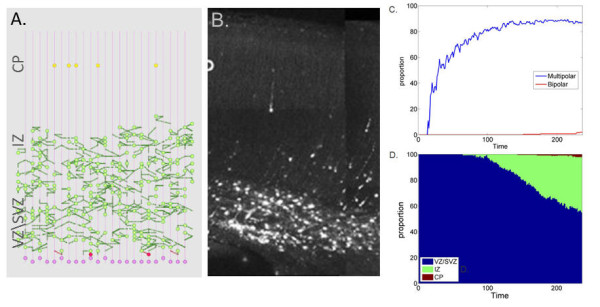
**Lis1 reduced activity experiment**. A. In-silico migration in Lis1-defective simulations. B. Histology of neuronal migration in the cortex of a Lis1 RNAi in mice (reproduced with permission from [24]). C. Distribution of multipolar (blue) and bipolar (red) neurons over time (averaged over 10 different simulations). D. Zone occupancy as function of time (VZ/SVZ blue), IZ (green) and CP (red) (averaged over 10 different simulations).

In simulations under reduced Lis1 activity, neuroblasts, once proliferated from the mother progenitor glial cell, entered the multipolar stage shortly after proliferation and dissociated from the glial fiber, rather than progressing normally along the fiber. Thus, reduced Lis1 activity shortens the duration over which neuroblasts remain associated with the glial fiber, and hastens multipolar migration. Consequently, the neuron population accumulated at the SVZ/VZ and the lower IZ and rarely migrated to the CP (Figure 4A and the movie in Additional File 2). This migration pattern that emerged in Lis1 reduced activity simulations concurs with the morphology observed in Lis1 RNAi in mice [21] in which neurons accumulate at the lower parts of the brain section closer to the VZ/SVZ (Figure 4B).

The population distribution in the Lis1 reduced activity simulations showed predominant multipolar cells that rarely adopt a bipolar state or enter the CP (Figure 4C,D). At the final stage of the simulation, the population consisted of 2% bipolar, 89% multipolar and 9% glial cells, of which 55% occupy the VZ/SVZ area, 43% the IZ, and 2% the CP. These results concur with experimentally measured distribution of the neuron population as 4% bipolar, 70% multipolar, 10% glial cell, (16% unclassified), and zone occupancies of 70%, 20% and 10% in VZ/SVZ, IZ and CP, respectively [24].

Under simulated conditions of reduced Lis1 activity, neurons lacking normal Lis1-activity responses to GABA showed altered duration and timing of association with the glial fiber, with subsequent cell accumulation at the SVZ and lower IZ areas. The emergent pattern in these simulations points to a possible direct role of Lis1-GABA interaction in the temporal progression of neuroblasts in the early stages of neuronal migration. This hypothesis could be tested experimentally by increasing GABA concentration in the cortex of animals with suppressed Lis1 activity. The higher GABA concentrations may boost Lis1 expression and restore normal association, possibly repairing the migration pattern in the reduced-expression strains.

### Lis1 reduced activity simulations predict an oscillatory neuron-glial association prior to the multipolar stage

**Figure 5:**
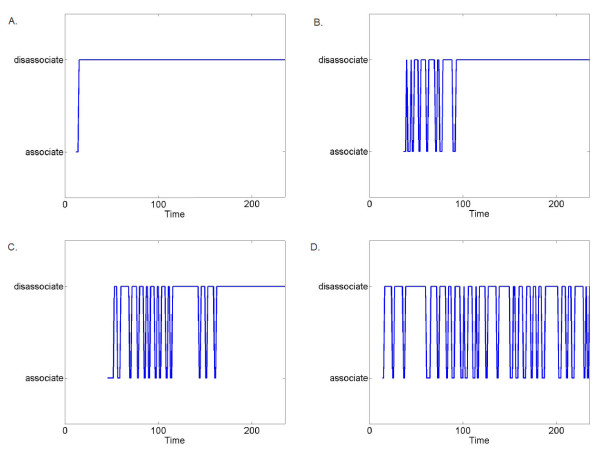
**Delay in multipolar migration in Lis1-defective neurons**. Oscillatory association-dissociation behavior of neurons from the glial fiber in four representative individual neurons. A. A cell that immediately adopts multipolar migration. B. Short delay. C. Long delay. D. Oscillatory association over the entire simulated period.

Analysis of the neuron-glial association in simulations of reduced Lis1 activity revealed an intriguing unforeseen phenomenon. We found that neuroblasts repeatedly attempt to associate with the glial fiber, displaying an oscillatory association and dissociation with the fiber, especially during the early stages of the neuron lifecycle. The oscillatory behavior began immediately following neuronal proliferation and lasted until completion of the multipolar migration stage. Thus the model, although not explicitly programmed to do so, produced a delay in the neuronal multipolar migration stage process (Figure 5 and the movie in Additional File 3).

The frequency and the duration of the oscillatory delay varied between individual neurons: 31% of the neurons began multipolar migration immediately after proliferation (Figure 5A) 58% had a delay of one embryonic day or less (Figure 5B) and 11% maintained this migratory behavior for longer than one embryonic day (Figure 5C), with 4% exhibiting this behavior over the entire simulated period (Figure 5D). Neither the oscillatory frequency nor the duration, were explicitly defined in the model. Rather, they emerged from the specified role of Lis1 and are promoted by the basal activity of the regulator.

The periodic association-disassociation phenomenon and the delay it forced in the multipolar migration could arise from contradicting instructions under reduced Lis1 activity. The neuroblasts received a weak signal to 'associate' from the basal level of Lis1 activity, while being instructed to 'migrate randomly' by the modified Lis1 activity.

We propose that the abnormal motility and defective neuronal division observed in Lis1 RNAi [21] emerge from a delay in the multipolar stage. This finding supports a hypothesis by which Lis1 plays a significant role in neuroblast development and cell motility [26]. These results point to a future experimental study to test the effect of the combination of timing and consequent spatial position in individual neuroblasts under reduced Lis1 activity conditions.

### Simulations under reduced DCX activity anticipate the aberrant migration observed in DCX RNAi rats and not the normal migration observed in DCX RNAi mice

**Figure 6:**
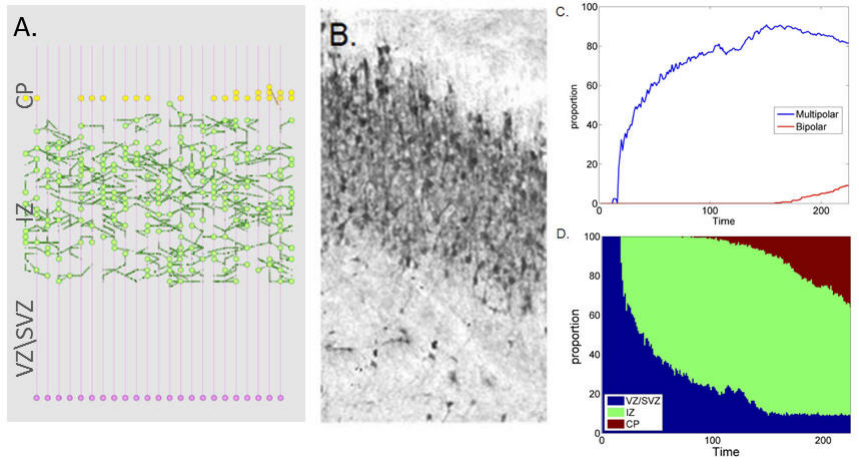
**DCX reduced activity experiment**. A. In-silico migration in DCX-defective simulations. B. Histology of neuronal migration in the cortex of rats in DCX RNAi (reproduced with permission from [31]). C. Distribution of multipolar (blue) and bipolar (red) neurons over time (averaged over 10 different simulations). D. Zone occupancy as function of time (VZ/SVZ blue), IZ (green) and CP (red) (averaged over 10 different simulations).

DCX function is highly complex and implicates various mechanisms. One of its possible functions is that it acts as a motility regulator that increases glial guided migration [2,27-30]. DCX RNAi rats show disruptions in radial migration, forming a subcortical band consisting of multipolar neurons in the IZ [31,32]. In our model, DCX acts as a motility regulator that stimulates re-association with the glial fiber in multipolar neurons. When neuronal ability to activate DCX is reduced to the basal level, re-association of multipolar neurons with the glial fiber is less likely to occur. Consequently, in simulations, the bipolar migration stage was largely halted, neurons did not advance from the IZ to the CP and reduced accumulation in the CP occured. Simulations under reduced DCX activity displayed accumulation of multipolar neurons at the upper end of the IZ closer to the CP. A minority of the neurons reacted to the random basal activity level and adopted the bipolar state, allowing them to enter the CP (Figure 6A and the movie in Additional File 4). The emergent pattern in reduced DCX activity simulations is consistent with observations that DCX RNAi rats develop a subcortical band of multipolar neurons [1,31,32] (Figure 6B).

Over the simulated period, multipolar neurons concentrated predominantly in the IZ, with only a small proportion (7%) adopting the bipolar state and entering the CP (Figure 6C-D). By the end of the simulation, the neuron occupancy in the different zones was in agreement with measurements from DCX RNAi rats [27] (numerical occupancies of 9%, 55% and 36% vs. experimentally observed occupancies of 10%, 60%, 30%, in the VZ/SVZ, IZ, and CP zones respectively). The final simulated neuron population comprised 9% glial cells, 82% multipolar stage cells and 9% bipolar stage cells. Though direct experimental measurements are not available for comparison, multipolar neuron population was increased and bipolar population was decreased in DXC RNAi rats, [30,33,34] supporting the simulation's results.

These results might explain the occurrence of DCX re-expression in DCX RNAi rats, with the resumption of normal bipolar migration [35]. When DCX is re-expressed, neurons are able to re-associate to the glial fiber, resulting in a normal bipolar morphology. Similarly, a highly bipolar morphology in DCX over-expression rats [27,31] could be explained by an intensive re-association of neurons that elevates the bipolar state.

### A single change in the way Lis1 and DCX guide motility may explain phenotypic variations between rats and mice

Studies investigating neuronal migration in Lis1 and DCX RNAi have typically focused on two model organisms, the rat and the mouse. Lis1 RNAi induced abnormal neuronal migration in both organisms [1]. In contrast, neuronal migration in DCX-RNAi animals showed a distinct phenotypic pattern: DCX RNAi in rats disrupted radial migration in the cortex [27,31], whereas DCX RNAi mice developed normally [31]. One possible hypothesis is that at the bipolar stage the mutual activity of Lis1 and DCX is different in the rat and the mouse. This hypothesis is supported by observations in Lis1 RNAi mice in which migration was largely restored under DCX over-expression [35], and concurs with previously suggested reciprocal functions and possible interaction between Lis1 and DCX [16,32,36].

In our model, reduced DCX activity generated an abnormal bipolar morphology consistent with that observed in DCX RNAi rats, rather than the normal morphology observed in the DCX RNAi mice [31]. DCX activity in the model is independent of Lis1 since both regulators are triggered autonomously in multipolar neurons. Thus Lis1 functioned normally in DCX reduced activity conditions, suggesting that Lis1 activity alone is not sufficient to promote normal bipolar migration.

These experimental observations combined with the model behavior led us to hypothesize that the rat cortex requires expression of both DCX and Lis1 for neurons to enter the bipolar migration stage. In contrast, bipolar morphology in the mouse cortex requires that neurons either normally express Lis1 or over-express DCX (Table 1). From this we predict that since the rat cortex requires expression of both Lis1 and DCX, higher expression of DCX in Lis1 RNAi rats will not restore normal migration as observed for Lis1-defective mice [36]. In addition, since Lis1 activity is sufficient to promote bipolar migration in mice, over-expression of DCX in this organism will have no effect on bipolar migration. This prediction allows for the possibility that DCX over-expression in mice enhances bipolar morphology as has been observed in rats [30,33,34] (Table 1).

**Table 1:** Bipolar migration under varying expression levels of Lis1 and DCX regulators

Lis1	DCX	Bipolar Migration (mouse cortex)	Bipolar Migration (rat cortex)
Reduced	Reduced	Reduced	Abnormal
Reduced	Normal	Reduced	Abnormal
Reduced	Over	Normal	Abnormal (*)
Normal	Reduced	Normal	Abnormal
Normal	Normal	Normal	Normal
Normal	Over	Normal/Enhanced (*)	Enhanced

Bipolar morphology under different expression levels of DCX and Lis1 in the mouse and rat cortex. The asterisk symbol indicates predicted morphologies.

## Conclusions

Our computational model of neuronal migration during brain development integrates a set of molecular sub-components that have been studied in isolation. In the model, cells are represented as executable objects whose migration is determined by explicit interactions between molecular entities defined and specified as a molecular 'program'. Overall simulated migration is thus an outcome of the collective molecular-level interactions in each object. In-silico experiments were conducted by changing parameters, enabling hypotheses and predictions about the process to be tested. This model is a first step in utilizing in-silico models and computational approaches for neuronal migration research.

The model described here focuses on the interplay between two major intracellular regulators, Lis1 and DCX, the glial fiber, and two extracellular guidance and motility cues, GABA and Reelin. While these are a subset of key molecular components, they nevertheless represent functional classes of mechanisms implicated in neuronal migration: guidance cues (GABA and the glial fiber), motility regulators (Lis1 and DCX), and stop signals (Reelin).

The model generated complex dynamics and behaviors of the migration process that are in qualitative agreement with the migratory process of cortical neurons in normal and abnormal conditions. Specifically, our model reproduced the distinct migration stages in the known cortex zones [37], bipolar and multipolar cell states over time [1,2], cell population dynamics [17,38], and specific disruptions of the migration induced by manipulations of the key regulators, Lis1 and DCX [24,27]. Furthermore, simulated condition of reduced Lis1 activity revealed that neuroblasts repeatedly associate and dissociate with the glial fiber before initiating a glial-independent multipolar migration. In addition, we hypothesized that a single change in the way neurons interpret the guidance cues can explain phenotypic variation between different organisms. These hypotheses could guide future experimental study.

We simplified the model for this study, deliberately omitting several aspects of migration. Obvious next steps would be to undertake more comprehensive modeling of the role of Lis1, DCX, GABA and Reelin, for example by a gradual activity. Similarly, the model could be extended to include (i) cell density within the different regions, (ii) neuron-neuron interaction, (iii) neuron-glial interaction, (iv) retarding forces encountered by migrating neurons, and (v) geometry of migrating neurons. Further elaborations might incorporate the role of migration factors we have excluded from the current study; for example, intracellular effectors known to influence neuronal migration such as CDK5 [39], signaling factors (e.g., Semaphorin-3A) [40] and calcium signaling [41]. Similarly, for the receptors implicated in migration, a possible refinement could incorporate in the model the effects of neuropilin-1 [40] and connexins [42].

More broadly, the neuronal migration model presented here highlights the potential for integrating theory, computational approaches and experimental data in neuroscience and in biology more generally [43-45]. Indeed, increasing focus is given to in-silico approaches for system-level analysis of the dynamics of complex biological systems (In-silico study of pancreatic morphogenesis and differentiation. Submitted; and [46-51]). We envision that in the long run, computational models will become important tools for understanding biological systems, for the identification of precise causes of abnormal development, and possibly disclose ways to restore normal development and function.

## Methods

### Setting up a computational platform

To compile the specifications of the model into an executable program (the Java source code of the model can be found in Additional File 5), we used MASON (Multi-Agent Simulator of Neighborhoods, http://cs.gmu.edu/~eclab/projects/mason/) coupled with Dynamic Data Display, a visualization tool developed by Microsoft Research (http://dynamicdatadisplay.codeplex.com/). MASON is an extendable, discrete event multi-agent simulation toolkit designed for multi-agent simulation tasks. This enables the model to simultaneously simulate operations and interactions of multiple agents, each operating a program using states and events that cause transitions between states, allowing the system to simultaneously drive several orthogonal states (designed in statecharts [52], using principles previously applied in [46]). Dynamic Data Display provides visualization of both current state and dynamic change from the previous state. The application uses linear interpolation algorithms to build intermediate values for the discrete states to visualize changes of the system over time. We implemented the platform in C# and used WPF library for User Interface purposes and data binding between the datasets and the animated objects. Visualization was coded using Microsoft DirectX 9 technology (SlimDX DirectX 9 managed wrapper; slimdx.org).

### Specifications of the neuronal migration model

**Figure 7:**
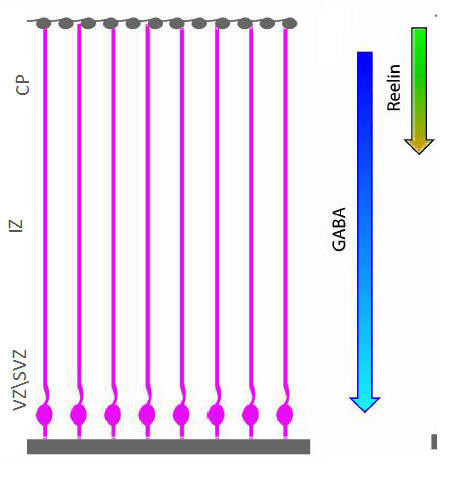
**Illustration of the simulated cortical cross-section**. Glial cells, whose nucleus positions lie at the bottom of the cross-section and their fiber is stretched to the surface of the cortex (of the cross-section). Reelin and GABA factors are present at densities that lie in a gradient from top to bottom of the cross-section.

**Figure 8:**
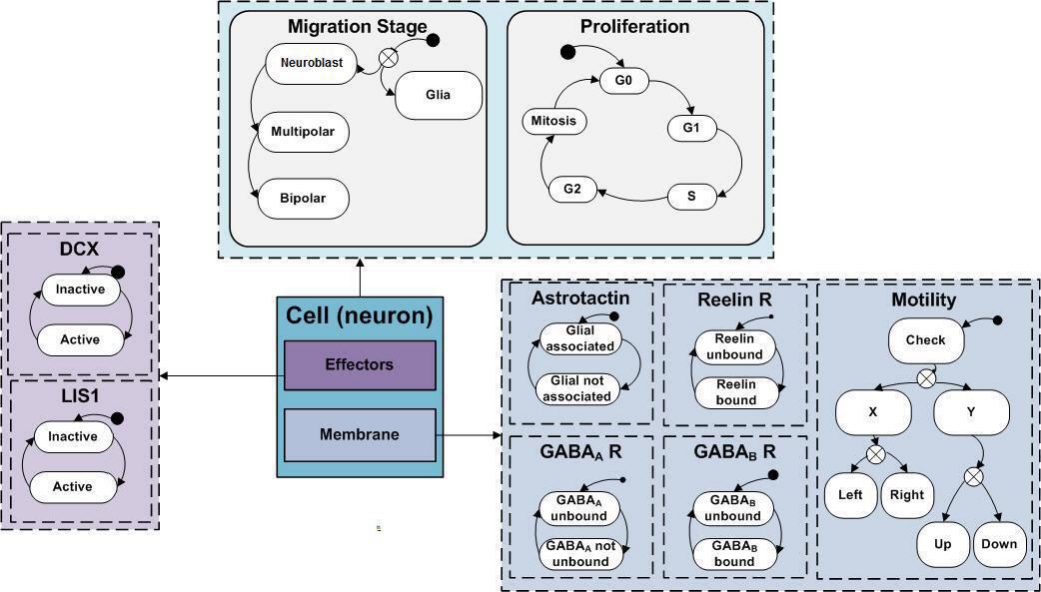
**Design of a neuron agent in the model**. Specifications of the model of a neuron as an agent in the computational model. Each agent consists of three objects, Effectors for intracellular regulation, Membrane for extra cellular signaling and Cell for developmental stages and proliferation. Each object specifies possible states for the element it covers. At run time, each orthogonal component (separated in dashed line) can be in one active state at a time.

A cortex cross-section is defined in the model as a 2D grid that overlays the agents and represents a cross section of the cortex. Glial fibers over the different cortical zones are represented by twenty-five vertically spanning routes (see illustration in Figure 7). The ventricular/subventricular, the intermediate zone and the cortical plate, each span a third of the cortical cross-section. The grid specifies concentrations for GABA and Reelin in a way that matches their concentrations in real cortex; that is, Reelin concentration is specified in the CP, and GABA concentration is specified as a gradient from the CP (top area of the grid) to the SVZ (bottom area of the grid). As an approximation, we assumed a linear gradient for GABA and Reelin degradation. A neuron is represented by an agent whose activity is governed by the specifications described diagrammatically in Figure 8. The neuron agent consists of three different objects, Cell, Membrane and Effectors. The Cell object comprises two components, Migration Stage and Proliferation. The Migration Stage component specifies possible migration stages for the agent. Two cell types, glial cells and migrating neurons, are distinguished by their specific roles in the simulation. Glial cells remain in the Glia state and proliferate, whereas neuron agents change their migration mode and evolve through the different developmental stages - Neuroblasts, Multipolar Stage and Bipolar Stage. The Proliferation component specifies five cell cycle phases G0, G1, S, G2 and Mitosis. Once a glial agent enters the Mitosis stage, a new agent is created and enters the Neuroblast state. The Effectors object serves as a cell- intrinsic regulation unit that specifies behaviors of two effectors: Lis1, DCX, each of which can be in an Active or Inactive state. The Membrane object serves as a cell-extrinsic signaling unit for the agent and consists of receptors that sense the cell's vicinity in the grid, and a motility unit that controls cell motion. The model has three components for the GABA_A_, GABA_B _and Reelin Receptors (which account for both VLDLR and ApoER2). Each receptor specifies activity in a binary fashion defined by an Unbound or Bound state. Similarly, the membrane specifies two states for the Astrotactin protein, Associated and Not Associated (with the glial fiber). The Motility component senses the close vicinity of the cells, continuously seeks possible moves and places the agent in a new position if applicable. The motion differs between agents in the states. Glial cells move vertically in four positions between the VZ and the SVZ during a cell cycle. Neuroblasts with glial-cell-associated Astrotactin migrate along the fiber. Multipolar neurons migrate randomly, whereas bipolar agents migrate vertically, provided that Reelin is not present. When Reelin is detected, the agent sets Astrotactin to Not Associated and calculates the nearest position at minimal distance from the IZ. To incorporate basal activity of regulators and receptors resulting from spontaneous intrinsic expression, we added noise to the transitions (i.e., a random element for transitions between the states). A cell may move or make a transition (e.g., from inactive to active) with a probability of 0.01. These transitions occur spontaneously in the absence of designated triggering events. We verified that the random element itself could not stimulate proper migration. Lis1- and DCX-defective simulations were implemented by erasing the Promote transition in the corresponding component; no additional constraints were forced upon the model. See the supplementary information in Additional File 6 for the result of a control experiment and the designs of the reduced activity in-silico experiments.

### Dynamic interactions in the simulation at run-time

Once the simulation executes, the cortex cross-section with 23 glial cells is initiated with 23 agents whose Migration Stage component is set to Glia and which are positioned at the bottom of the grid (VZ). The Proliferation component of these cells continuously moves over the cell cycle stages and concurrently, the motion unit moves the agent vertically over the four bottom fiber indexes in the grid. When the agent enters the Mitosis stage, a new agent, whose state is set to Neuroblast is created. As the proliferation terminates, the Motility Unit places the glial cell in the initial position for G0 phase. Initially, the Effectors of the new agent are in an Inactive state. GABA_A_, GABA_B _and Reelin Receptors are set to Unbound and the Astrotactin is in the Associated state. As the simulation advances, the Motion Unit of the agent directs its migration radially over the glial fiber indexes in the grid. As the neuron moves along the glial fiber, it detects GABA concentration and sets the state of the GABA_A _receptor to Bound. In turn, the cell promotes the Lis1 effector, which enters the Active state to maintain normal association with the fiber. On entry into the intermediate zone, Lis1 activity is reduced as the receptor loses its activity and moves its state to Unbound. In turn, the cell sets its state to Multipolar, the Astrotactin state to Not Associated and the motility unit directs random movement over the 2D space. When the GABA_B _receptor detects the higher GABA concentrations (i.e., the values in the grid beyond a predefined threshold) in the middle grid area (IZ zone), it sets its state to Bound and consequently sets the state of DCX and Lis1 to Active. The neuron then enters the Bipolar state, its Astrotactin becomes Associated and the Motility Unit directs a radial motion. Finally, when the Reelin receptors component detects Reelin in the cortex cross-section, its state is set to Bound, the Astrotactin moves into the Not Associated state and the Motility Unit searches for an optimal location to accumulate.

### Mathematical analysis of the simulation outputs

To determine multipolar and bipolar stage of agents from the path formed at a specific time point, we employ the motion directionality analysis in [25] as follows:

(1) $$J=\frac{d}{l},$$

where $d=\sqrt{{\left(x\left(t_{k+1}\right)-x\left(t_{k}\right)\right)}^{2}+{\left(y\left(t_{k+1}\right)-y\left(t_{k}\right)\right)}^{2}}$ is the net displacement during a time subdivision (*t*_*k*_), (*t*_*k*__+1_) ⊆ (0, *t*_*n*_) for which the neuron preserves the same migration stage, where *t*_*n *_is the final time point of the simulation, *0 < k < n*, and $l=T\left(<u_{\vec{x}}{>}^{2}+<u_{\vec{y}}{>}^{2}\right)$ (for a time interval *T*) is the length of the neuron path. The index J scores values in the range between 0 and 1 to specify directionality of an agent motion (0 for complete randomness and 1 for movement along a straight line). This parameter quantifies the amount of entropy in the movement of a neuron by comparing the length of the total path of the cell between two different time points and the straight distance between them. It assesses whether the movement of the cell lean toward deterministic or random. This parameter can therefore be used to distinguish between the more directed bipolar movement and the more random multipolar migration. Therefore, the higher the *J*-index score, the more likely the neuron is bipolar, and vice versa; the lower the score, the more likely the neuron is multipolar. The *J*-index threshold that distinguishes multipolar from bipolar was determined by an optimization algorithm aimed at maximizing agreement with the multipolar population data at three time points: 1/3, 2/3 and end of simulation. The optimization approach uses a simulated annealing algorithm composed of successive trials, each of which sets new thresholds as the arithmetic mean of the two optimal previously computed thresholds, with noise insertion to avoid local maxima. The optimization yielded a range of threshold values 0.2 to 0.4 inclusive, from which we set the threshold to be 0.25 as in [25]. The velocities of neurons are computed for each time interval (*t*_*k*_), (*t*_*k*__+1_) ⊆ (0, *t*_*n*_), as $\frac{dx}{dt}$ i.e., the ratio of the path length for the defined time interval to the length of the respective time interval:

$$\begin{array}{ccc} n_{x}\left(t_{k+1}\right)=\frac{x_{t_{k+1}}-x_{t_{k}}}{t_{k+1}-t_{k}} & \text{and} & n_{y}\left(t_{k+1}\right)=\frac{y_{t_{k+1}}-y_{t_{k}}}{t_{k+1}-t_{k}} \end{array}$$

With *n*_*x *_and *n*_*y *_being the horizontal and vertical velocity components and $x_{t_{i}}$, $y_{t_{i}}$ being the positions of the neuron at time *t*_*i*_, *0 < i < n *; *t*_*n *_is the final time point of the simulation and *0 < k < n*.

## Competing interests

The authors declare that they have no competing interests.

## Authors' contributions

YS conceived and coordinated the study, designed the computational model and the in-silico experiments, analyzed the data and produced the figures. CCC carried out the in-silico simulations. NS built the visualization platform. MS and DK carried out the mathematical analysis in Figure 3C and Figure 3D. YS, DK and SE wrote the paper. All authors have read and approved the final manuscript.

## Supplementary Material

Additional file 1 **Recorded simulation of neuronal migration under normal conditions**. Click here for file

Additional file 2 **Recorded simulation of neuronal migration under reduced Lis1 activity**. Click here for file

Additional file 3 **Oscillatory association-dissociation behavior of four representative neurons**. Neurons are labeled green when they are disassociated from the glial fiber and change their color to red upon association. Click here for file

Additional file 4 **Recorded simulation of neuronal migration under reduced DCX activity**. Click here for file

Additional file 5 **A compressed file of the source code of the model. Written in Java and compiled using Mason (see Material and Methods)**. Click here for file

Additional file 6 **Supplementary information: a control experiments and the design of the reduced activity in-silico experiments**. Click here for file

## References

[B1] LoTurco JJ Bai J The multipolar stage and disruptions in neuronal migration Trends Neurosci 2006 29 7 407 413 10.1016/j.tins.2006.05.006 16713637

[B2] Ayala R Shu T Tsai LH Trekking across the brain: the journey of neuronal migration Cell 2007 128 1 29 43 10.1016/j.cell.2006.12.021 17218253

[B3] Rice DS Curran T Role of the reelin signaling pathway in central nervous system development Annu Rev Neurosci 2001 24 1005 1039 10.1146/annurev.neuro.24.1.1005 11520926

[B4] Trommsdorff M Gotthardt M Hiesberger T Shelton J Stockinger W Nimpf J Hammer RE Richardson JA Herz J Reeler/Disabled-like disruption of neuronal migration in knockout mice lacking the VLDL receptor and ApoE receptor 2 Cell 1999 97 6 689 701 10.1016/S0092-8674(00)80782-5 10380922

[B5] Zheng W Yuan X Guidance of cortical radial migration by gradient of diffusible factors Cell Adh Migr 2008 2 1 48 50 10.4161/cam.2.1.6001 19262126 PMC2635003

[B6] Zubler F Douglas R A framework for modeling the growth and development of neurons and networks Front Comput Neurosci 2009 3 25 19949465 10.3389/neuro.10.025.2009 PMC2784082

[B7] Behar TN Schaffner AE Scott CA Greene CL Barker JL GABA receptor antagonists modulate postmitotic cell migration in slice cultures of embryonic rat cortex Cereb Cortex 2000 10 9 899 909 10.1093/cercor/10.9.899 10982750

[B8] Forster E Tielsch A Saum B Weiss KH Johanssen C Graus-Porta D Muller U Frotscher M Reelin, Disabled 1, and beta 1 integrins are required for the formation of the radial glial scaffold in the hippocampus Proc Natl Acad Sci USA 2002 99 20 13178 13183 10.1073/pnas.202035899 12244214 PMC130606

[B9] Franco SJ Martinez-Garay I Gil-Sanz C Harkins-Perry SR Muller U Reelin regulates cadherin function via Dab1/Rap1 to control neuronal migration and lamination in the neocortex Neuron 2011 69 3 482 497 10.1016/j.neuron.2011.01.003 21315259 PMC3056352

[B10] Meyer G Goffinet AM Fairen A What is a Cajal-Retzius cell? A reassessment of a classical cell type based on recent observations in the developing neocortex Cereb Cortex 1999 9 8 765 775 10.1093/cercor/9.8.765 10600995

[B11] Hack I Bancila M Loulier K Carroll P Cremer H Reelin is a detachment signal in tangential chain-migration during postnatal neurogenesis Nat Neurosci 2002 5 10 939 945 10.1038/nn923 12244323

[B12] Horesh D Sapir T Francis F Wolf SG Caspi M Elbaum M Chelly J Reiner O Doublecortin, a stabilizer of microtubules Hum Mol Genet 1999 8 9 1599 1610 10.1093/hmg/8.9.1599 10441322

[B13] Kappeler C Saillour Y Baudoin JP Tuy FP Alvarez C Houbron C Gaspar P Hamard G Chelly J Metin C Branching and nucleokinesis defects in migrating interneurons derived from doublecortin knockout mice Hum Mol Genet 2006 15 9 1387 1400 10.1093/hmg/ddl062 16571605

[B14] Pramparo T Youn YH Yingling J Hirotsune S Wynshaw-Boris A Novel embryonic neuronal migration and proliferation defects in Dcx mutant mice are exacerbated by Lis1 reduction J Neurosci 2010 30 8 3002 3012 10.1523/JNEUROSCI.4851-09.2010 20181597 PMC2861429

[B15] Hippenmeyer S Youn YH Moon HM Miyamichi K Zong H Wynshaw-Boris A Luo L Genetic mosaic dissection of Lis1 and Ndel1 in neuronal migration Neuron 2010 68 4 695 709 10.1016/j.neuron.2010.09.027 21092859 PMC3044607

[B16] Caspi M Atlas R Kantor A Sapir T Reiner O Interaction between LIS1 and doublecortin, two lissencephaly gene products Hum Mol Genet 2000 9 15 2205 2213 11001923 10.1093/oxfordjournals.hmg.a018911

[B17] Kriegstein AR Noctor SC Patterns of neuronal migration in the embryonic cortex Trends Neurosci 2004 27 7 392 399 10.1016/j.tins.2004.05.001 15219738

[B18] Gleeson JG Classical lissencephaly and double cortex (subcortical band heterotopia): LIS1 and doublecortin Curr Opin Neurol 2000 13 2 121 125 10.1097/00019052-200004000-00002 10987567

[B19] Fishell G Hatten ME Astrotactin provides a receptor system for CNS neuronal migration Development 1991 113 3 755 765 1821847 10.1242/dev.113.3.755

[B20] Meyer G Goffinet AM Prenatal development of reelin-immunoreactive neurons in the human neocortex J Comp Neurol 1998 397 1 29 40 10.1002/(SICI)1096-9861(19980720)397:1<29::AID-CNE3>3.0.CO;2-K 9671277

[B21] Tsai JW Bremner KH Vallee RB Dual subcellular roles for LIS1 and dynein in radial neuronal migration in live brain tissue Nat Neurosci 2007 10 8 970 979 10.1038/nn1934 17618279

[B22] Williams SN Locke CJ Braden AL Caldwell KA Caldwell GA Epileptic-like convulsions associated with LIS-1 in the cytoskeletal control of neurotransmitter signaling in Caenorhabditis elegans Hum Mol Genet 2004 13 18 2043 2059 10.1093/hmg/ddh209 15254012

[B23] Tabata H Nakajima K Multipolar migration: the third mode of radial neuronal migration in the developing cerebral cortex J Neurosci 2003 23 31 9996 10001 14602813 10.1523/JNEUROSCI.23-31-09996.2003 PMC6740853

[B24] Tsai JW Chen Y Kriegstein AR Vallee RB LIS1 RNA interference blocks neural stem cell division, morphogenesis, and motility at multiple stages J Cell Biol 2005 170 6 935 945 10.1083/jcb.200505166 16144905 PMC2171430

[B25] Distasi C Ariano P Zamburlin P Ferraro M In vitro analysis of neuron-glial cell interactions during cellular migration Eur Biophys J 2002 31 2 81 88 10.1007/s00249-001-0194-y 12012111

[B26] Vallee RB Tsai JW The cellular roles of the lissencephaly gene LIS1, and what they tell us about brain development Genes Dev 2006 20 11 1384 1393 10.1101/gad.1417206 16751177

[B27] Bai J Ramos RL Ackman JB Thomas AM Lee RV LoTurco JJ RNAi reveals doublecortin is required for radial migration in rat neocortex Nat Neurosci 2003 6 12 1277 1283 10.1038/nn1153 14625554

[B28] Reiner O Gdalyahu A Ghosh I Levy T Sapoznik S Nir R Sapir T DCX's phosphorylation by not just another kinase (JNK) Cell Cycle 2004 3 6 747 751 15118415

[B29] Koizumi H Tanaka T Gleeson JG Doublecortin-like kinase functions with doublecortin to mediate fiber tract decussation and neuronal migration Neuron 2006 49 1 55 66 10.1016/j.neuron.2005.10.040 16387639

[B30] LoTurco J Doublecortin and a tale of two serines Neuron 2004 41 2 175 177 10.1016/S0896-6273(04)00006-6 14741096

[B31] Ramos RL Bai J LoTurco JJ Heterotopia formation in rat but not mouse neocortex after RNA interference knockdown of DCX Cereb Cortex 2006 16 9 1323 1331 16292002 10.1093/cercor/bhj074

[B32] Bai J Ramos RL Paramasivam M Siddiqi F Ackman JB LoTurco JJ The role of DCX and LIS1 in migration through the lateral cortical stream of developing forebrain Dev Neurosci 2008 30 1-3 144 156 10.1159/000109859 18075262

[B33] Bielas SL Gleeson JG Cytoskeletal-associated proteins in the migration of cortical neurons J Neurobiol 2004 58 1 149 159 10.1002/neu.10280 14598377

[B34] Schaar BT Kinoshita K McConnell SK Doublecortin microtubule affinity is regulated by a balance of kinase and phosphatase activity at the leading edge of migrating neurons Neuron 2004 41 2 203 213 10.1016/S0896-6273(03)00843-2 14741102

[B35] Manent JB Wang Y Chang Y Paramasivam M LoTurco JJ Dcx reexpression reduces subcortical band heterotopia and seizure threshold in an animal model of neuronal migration disorder Nat Med 2009 15 1 84 90 10.1038/nm.1897 19098909 PMC2715867

[B36] Tanaka T Serneo FF Higgins C Gambello MJ Wynshaw-Boris A Gleeson JG Lis1 and doublecortin function with dynein to mediate coupling of the nucleus to the centrosome in neuronal migration J Cell Biol 2004 165 5 709 721 10.1083/jcb.200309025 15173193 PMC2172383

[B37] Noctor SC Martinez-Cerdeno V Ivic L Kriegstein AR Cortical neurons arise in symmetric and asymmetric division zones and migrate through specific phases Nat Neurosci 2004 7 2 136 144 10.1038/nn1172 14703572

[B38] Edmondson JC Liem RK Kuster JE Hatten ME Astrotactin: a novel neuronal cell surface antigen that mediates neuron-astroglial interactions in cerebellar microcultures J Cell Biol 1988 106 2 505 517 10.1083/jcb.106.2.505 3276720 PMC2114977

[B39] Zukerberg LR Patrick GN Nikolic M Humbert S Wu CL Lanier LM Gertler FB Vidal M Van Etten RA Tsai LH Cables links Cdk5 and c-Abl and facilitates Cdk5 tyrosine phosphorylation, kinase upregulation, and neurite outgrowth Neuron 2000 26 3 633 646 10.1016/S0896-6273(00)81200-3 10896159

[B40] Chen G Sima J Jin M Wang KY Xue XJ Zheng W Ding YQ Yuan XB Semaphorin-3A guides radial migration of cortical neurons during development Nat Neurosci 2008 11 1 36 44 10.1038/nn2018 18059265

[B41] Weissman TA Riquelme PA Ivic L Flint AC Kriegstein AR Calcium waves propagate through radial glial cells and modulate proliferation in the developing neocortex Neuron 2004 43 5 647 661 10.1016/j.neuron.2004.08.015 15339647

[B42] Elias LA Wang DD Kriegstein AR Gap junction adhesion is necessary for radial migration in the neocortex Nature 2007 448 7156 901 907 10.1038/nature06063 17713529

[B43] Edelman LB Chandrasekaran S Price ND Systems biology of embryogenesis Reprod Fertil Dev 2010 22 1 98 105 10.1071/RD09215 20003850 PMC2921326

[B44] Palsson B The challenges of in silico biology Nat Biotechnol 2000 18 11 1147 1150 10.1038/81125 11062431

[B45] van Ooyen A Using theoretical models to analyse neural development Nat Rev Neurosci 2011 12 6 311 326 10.1038/nrn3031 21587288

[B46] Setty Y Cohen IR Dor Y Harel D Four-dimensional realistic modeling of pancreatic organogenesis Proc Natl Acad Sci USA 2008 105 51 20374 20379 10.1073/pnas.0808725105 19091945 PMC2629264

[B47] Kugler H Larjo A Harel D Biocharts: a visual formalism for complex biological systems J R Soc Interface 2010 7 48 1015 1024 10.1098/rsif.2009.0457 20022895 PMC2880074

[B48] Kam N Kugler H Marelly R Appleby L Fisher J Pnueli A Harel D Stern MJ Hubbard EJ A scenario-based approach to modeling development: a prototype model of C. elegans vulval fate specification Dev Biol 2008 323 1 1 5 10.1016/j.ydbio.2008.07.030 18706404 PMC2949293

[B49] Wang DY Cardelli L Phillips A Piterman N Fisher J Computational modeling of the EGFR network elucidates control mechanisms regulating signal dynamics BMC Syst Biol 2009 3 118 10.1186/1752-0509-3-118 20028552 PMC2807436

[B50] Fisher J Henzinger TA Executable cell biology Nat Biotechnol 2007 25 11 1239 1249 10.1038/nbt1356 17989686

[B51] Setty Y Dalfó D Korta DZ Hubbard EJA Kugler H A model of stem cell population dynamics: in-silico analysis and in-vivo validation Development 2011 in press 10.1242/dev.067512 PMC3231771 22147952

[B52] Harel D Statecharts: A visual formalism for complex systems Sci Comput Program 1987 8 3 231 274 10.1016/0167-6423(87)90035-9

[B53] Yokota Y Gashghaei HT Han C Watson H Campbell KJ Anton ES Radial glial dependent and independent dynamics of interneuronal migration in the developing cerebral cortex PLoS One 2007 2 8 e794 10.1371/journal.pone.0000794 17726524 PMC1950908

