# Supplementary material for: How neurons migrate: a dynamic in-silico model of neuronal migration in the developing cortex
Source: BMC Syst Biol. 2011 Sep 30;5:154. doi: 10.1186/1752-0509-5-154 (PMC3198702; doi:10.1186/1752-0509-5-154)
Supplement: Additional file 6 — Supplementary information: a control experiments and the design of the reduced activity in-silico experiments. [file 1752-0509-5-154-S6.DOC]

**How neurons migrate: a dynamic in-silico model of neuronal migration in the developing cortex**

**Yaki Setty^1,2*^, Chih-Chun Chen^3^, Maria Secrier^4^, Nikita Skoblov^5^, Dimitrios Kalamatianos^6^, Stephen Emmott^1^**

^1^ Computational Science Laboratory, Microsoft Research, Cambridge, CB3 0FB, UK.

^2^ Department of Computer Science and applied Mathematics, Weizmann Institute of Science, Rehovot, Israel

^3^ Centre National de la Recherche Scientifique, Paris, France.

^4^ European Molecular Biology Laboratory, Heidelberg, Germany.

^5^ The Faculty of Computational Mathematics and Cybernetics, Moscow State University, Moscow, Russia.

^6^ The Hamilton Institute, National University of Ireland, Maynooth, Ireland

*Corresponding author

Email addresses:

YS: [yaki.setty@gmail.com](mailto:yaki.setty@gmail.com)

CCC: [chihchun_chen@yahoo.co.uk](mailto:yaki.setty@gmail.com)

MS: [maria.secrier@embl.de](mailto:maria.secrier@embl.de)

NS: [mtgrhox@gmail.com](mailto:mtgrhox@gmail.com)

DK: [dimitris.kalamatianos@nuim.ie](mailto:dimitris.kalamatianos@nuim.ie)

SE: [semmott@microsoft.com](mailto:semmott@microsoft.com)

Correspondence should be addressed to:
Dr. Yaki Setty, [yaki.setty@gmail.com](mailto:yaki.setty@gmail.com)

**Supplementary Information**

**A control experiment**

To test that the random activities of cells cannot stimulate proper migration, we rendered the cells inactive by disabling the transitions in the simulation , leaving only the random activity. Under these constraints neurons lacked the ability to take action in response to cell extrinsic signaling cues or intrinsic regulation, rather they acted based on the basal level of activation in the random component. The migration in these simulations consisted of inactive neurons that proliferate from the glial cell but lacked proper differentiation and migration behaviors. Consequently the inert neurons accumulated at the VZ area close to the glial mother cells (Supplemental Figure  [1](#page1)). These results confirmed that the basal level of activity of the factors and the random components in the molecular program cannot stimulate proper migration on their own. Furthermore, the results showed that the migration patterns in the simulations are a consequence of the precise deterministic causality of interactions over the molecular network.


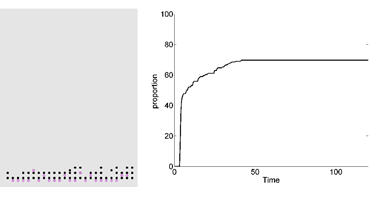


Supplemental Figure 1: neuronal migration in a control stochastic program. Left. The emergent simulation (inactive neuroblasts (black) glial cells (pink)). Right. Population distribution over time.


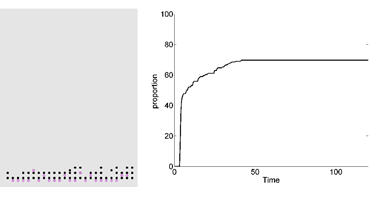


**Designs diagrams for reduced activity experiments**


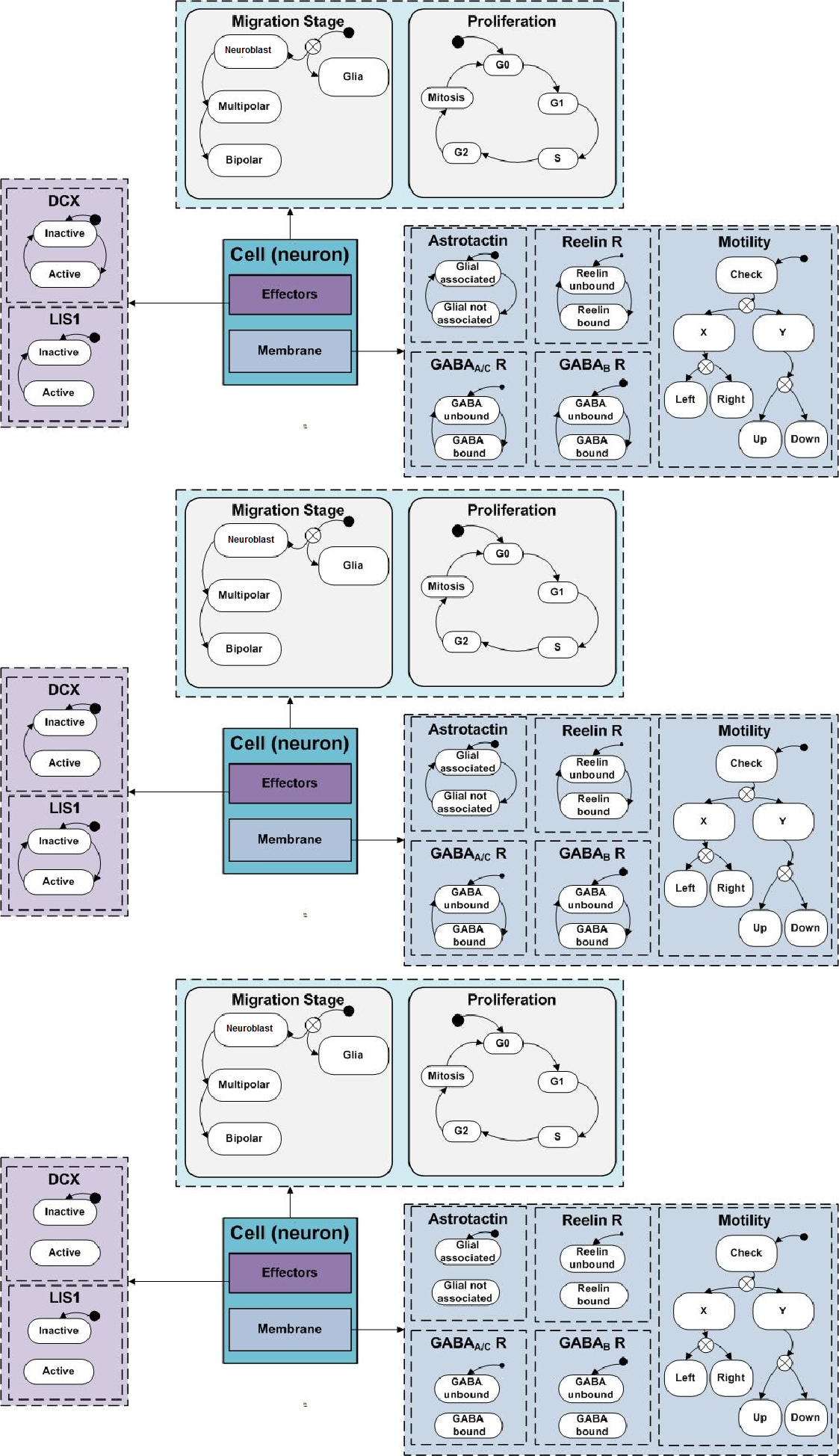


Supplemental Figure 2: Design diagrams for reduced activity of factors in the simulation. Top: Lis1 reduced activity. Middle: DCX reduced activity. Bottom: Control stochastic design (reduced activity of all molecular factors).
